# Implementing large scale fast track diagnostics in a comprehensive cancer center, pre- and post-measurement data

**DOI:** 10.1186/s12913-018-2868-5

**Published:** 2018-02-07

**Authors:** W. H. van Harten, N. Goedbloed, A. H. Boekhout, S. Heintzbergen

**Affiliations:** The Netherlands Cancer Institute, Plesmanlaan 121, 1066CX, Amsterdam, The Netherlands

**Keywords:** Patient centered care, Patient logistics, Early cancer detection, Cancer care facilities, Critical pathways, Health services, Oncology service hospital, Organizational, Organizational management, Operations management

## Abstract

**Background:**

In general, patients with a cancer suspicion visit the hospital multiple times before diagnosis is completed. Using various “operations management” techniques a few fast track diagnostic services were implemented in the Netherlands Cancer Institute (NKI) in 2006. Growing patient numbers and increasing process complexity, led to diminished service levels. To decrease the amount of patient visits and to extend these services beyond the (obvious) breast cancer services, fast track diagnostics is now implemented for all 18 cancer types that present with a frequency of minimally one per week.

**Methods:**

The throughput time (first visit to diagnosis conversation) was measured before, and after implementation of fast track diagnostics. The process was redesigned closely involving the multidisciplinary teams. In an eclectic approach elements from lean management, theory of constraints and mathematical analysis were used to organize slots per tumor type for MRI, CT, PET and echography. A post measurement was performed after 3 and 6 months.

**Results:**

In pre measurement access time was calculated to be 10 to 15 workdays, mean throughput time was 6.0 workdays. It proved possible to design the process of 18 tumors as a fast track, of which 7 as “one stop shop” (diagnosis completed in one visit). Involvement of clinical- and board leadership, massive communication efforts and commitment of physicians to reschedule their work proved decisive. After 3 and 6 months of implementation, the mean access time was 8.2 and 8.7 workdays respectively and mean throughput time was 3.4 and 3.3 workdays respectively.

**Conclusions:**

Throughput- and access time were considerably shortened after implementation of fast track diagnostics for 18 cancer types. The involvement of physicians in reorganizing their work and rapid responding to their needs during the implementation phase were a crucial success factor.

## Background

With the increasing incidence of cancer and the introduction of early detection and screening programs, the numbers of patients presenting to be diagnosed is growing. The tendency to concentrate cancer services, in view of quality criteria, use of expensive infrastructure and minimum numbers related to volume-outcome discussions, adds to this trend. The introduction of managed competition-like systems urge hospitals to compete both patient centeredness and efficiency [1, 2]. In response institutions explore innovative approaches to redesign their services, for instance by using techniques that are derived from the business domain or Operations Management [3–7]. International examples are mostly reported on single tumor services, such as sarcoma [8], brain- [9] and head and neck cancer [10, 11].

Patients with a cancer suspicion often visit the hospital multiple times before the diagnosis is completed. In operations management literature we can find various papers referring to service improvement using redesign or lean management techniques [12], but so far little evidence is found on large scale- or multiple service improvement, especially in the hospital environment. This is especially relevant as, at least theoretically, improvement in isolated services by allocating fast track slots, can lead to suboptimalization elsewhere in the organization. Reports on isolated services, such as breast cancer in oncology are available, but papers on large scale improvement interventions are rather scarce and not found on cancer services.

Using various “operations management” techniques a limited number of fast track diagnostic services were implemented in the Netherlands Cancer Institute (NKI) in 2006. Initially, predominantly fast track diagnostics in breast cancer was provided and later a few more tumor types were added. Increasing process complexity especially due to additional diagnostic modalities, fast growing patient numbers and numbers of clinical trials led to diminished service levels; in about 7 years the number of contacts related to new outpatients almost doubled. From benchmarks [7, 13] as well as feedback from radiology diagnostic companies, we knew that there was already a relatively high degree of efficiency in capacity use and hardly any redundancy. The organization decided to embark on a project to improve the quality and efficiency of all outpatient services for those cancer types that provided diagnostics for at least 50 new patients with the same (suspected) tumor diagnosis per year. Diagnosis is defined as pathological diagnosis including tumor classification and treatment plan proposal. The objective was to introduce fast track diagnostics for the identified 18 cancer types, and decrease the access and throughput time, with no- or as limited capacity extension as possible. For patients this was defined as decreasing the amount of patient visits and the period of uncertainty to a minimum, preferably one day. In popular terms: reduction of “sleepless nights” for patients under suspicion of a cancer diagnosis. We report on the process of analysis, redesign, implementation, organizational dynamics and first results using a pre and post measurement design.

## Methods

**Fig. 1:**
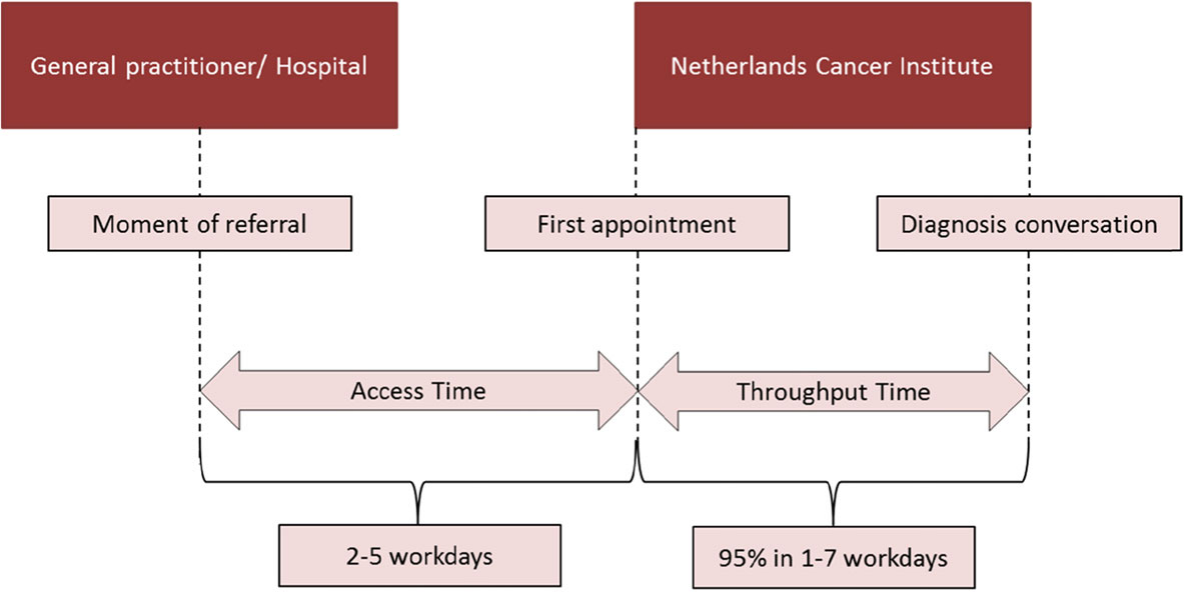
The access time (date the appointment was made to first face to face contact), and throughput time (first face to face contact to consultation in which diagnosis and first treatment advice is provided), was measured before redesigning the diagnosis process, and repeated after implementation of fast track diagnostics

Figure 1 a steering group was formed, consisting of clinical leaders and senior management, to guide the various process steps and show leadership commitment. The project team consisted of internal project staff and two external consultants all experienced in operations improvement. For every tumor pathway, a responsible staff in terms of content (“medical pathway owner”) and organizational issues (“process pathway owner”) was identified; these were not necessarily the same.

### Analysis

The access time (date the appointment was made to first face to face contact), and throughput time (first face to face contact to consultation in which diagnosis and first treatment advice is provided), was measured before redesigning the diagnosis process, and repeated after implementation of fast track diagnostics. In the pre measurement throughput time was retrospectively measured for 175 patients and 10 cancer types; we involved a group of patients who received fast track diagnostics, as introduced in 2006, from October to December 2011. The diagnostic process for 18 cancer types was further analyzed by the project team in close corporation with all involved tumor boards. It consisted of a quantitative and qualitative process analysis involving input and process variation, slot use and constraints for all direct and indirect processes in the diagnostics. Since no details on individuals were reported, no written informed consent was obtained.

### Redesign

In the redesign phase as first step, in close cooperation with the multidisciplinary teams including the supporting staff, an optimization proposal was drafted. In an eclectic approach elements from lean management, theory of constraints and mathematical analysis were used to design and organize reserved slots for Magnetic Resonance Imaging (MRI), Computed Tomography (CT), Positron Emission Tomography (PET) and echography related to the fast track outpatient capacity. Lean management was especially used for reducing waste, such as duplications in diagnostics as a consequence of ineffective referral procedures and unnecessary delay in decisions on the diagnostic package to be applied. The Theory of Constraints was used to identify and clear the main bottlenecks in the diagnostic process; this referred for instance to low frequencies of multidisciplinary team conferences and specific time periods on a few days per week where accumulation of diagnostic orders occurred. These were used as starting point from which the diagnosis process was redesigned. With mathematical analysis expected number of fast track diagnostic patients and associated needed capacity was calculated using recent patient data.

### Implementation

After agreement of both the steering group and the various tumor boards this phase was started. The implementation of the redesign was executed according to a predefined plan per diagnostic pathway per tumor, using an inventory of barriers and facilitators, active involvement of “pathway owners” and close supervision and support for every redesigned track to be implemented. The dedicated project officers with the team of “process pathway owners” were charged with the responsibility for implementation and communication with multidisciplinary team members.

### Evaluation

We developed a 14 items digital satisfaction questionnaire especially focusing on patient experiences with the diagnostic process. In order to evaluate the performance and to enable management to further assure the service levels in view of underperformance or variations in patient inflow, a permanent monitoring system was designed. A first post measurement after 3 and 6 months will be reported upon.

Where relevant we will report on the main sociodynamic aspects that interfered with- or were helpful in a smooth execution of the project.

## Results

Access time is estimated to be 10 to 15 workdays in pre measurement. In the diagnostic phase we measured a pilot series of 175 patients and 10 cancer types to raise awareness among staffs and provide a base measurement for the project. It proved that the throughput time ranged between 2 workdays onwards to 58 (mean throughput time 6.0 workdays) and the numbers of visits ranged from 1 to 12 per patient (Table 1). It has to be taken into account that the all over average score was positively influenced by the breast cancer pathway’s performance, as this relates to relatively large numbers.

**Table 1:** Base measurement

	Number of patients	Mean throughput time (workdays)	Mean number of hospital visits
Colon/Rectum	5	12,4	3
Breast	125	3,0	1,4
Head and Neck	16	17,2	4,6
Bladder	9	17,6	4,1
Prostate	12	11,3	2,3
Total	167	6,0	2,0

In Table 1 cervix (*n* = 3), endometrium (*n* = 2), vulva (*n* = 1), ovary (*n* = 1) and esophagus/stomach (*n* = 1) results were not presented due to small samples.

### Redesign

The base measurement data were used as input for discussions in all involved tumor boards; first to organize awareness and commitment, second to start the analytic phase in which bottlenecks and wasteful activities were identified and lastly to redesign the process in such a way that the fastest possible diagnostic track was defined, preferably on the same day (one stop shop), unless imperative reasons forced us to deviate from that objective.

To enable fast access, a digital referral procedure was implemented involving a formatted referral and a prescreening of every possibly eligible patient by a mandated physician per tumor service.

**Fig. 2:**
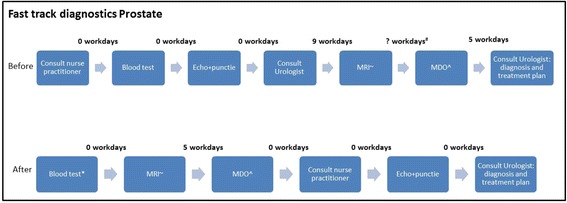
Mean throughput time in prostate diagnostics before and after fast track diagnostics implementation. ^MDO= multidisciplinary physicians meeting. ~MRI=magnetic resonance imaging. # Multidisciplinary physicians meeting was not registered in baseline measurement (estimated time between MRI and MDO is 3 workdays). *In patients without MRI indication a blood test is done before consult physician assistant (same day)

**Fig. 3:**
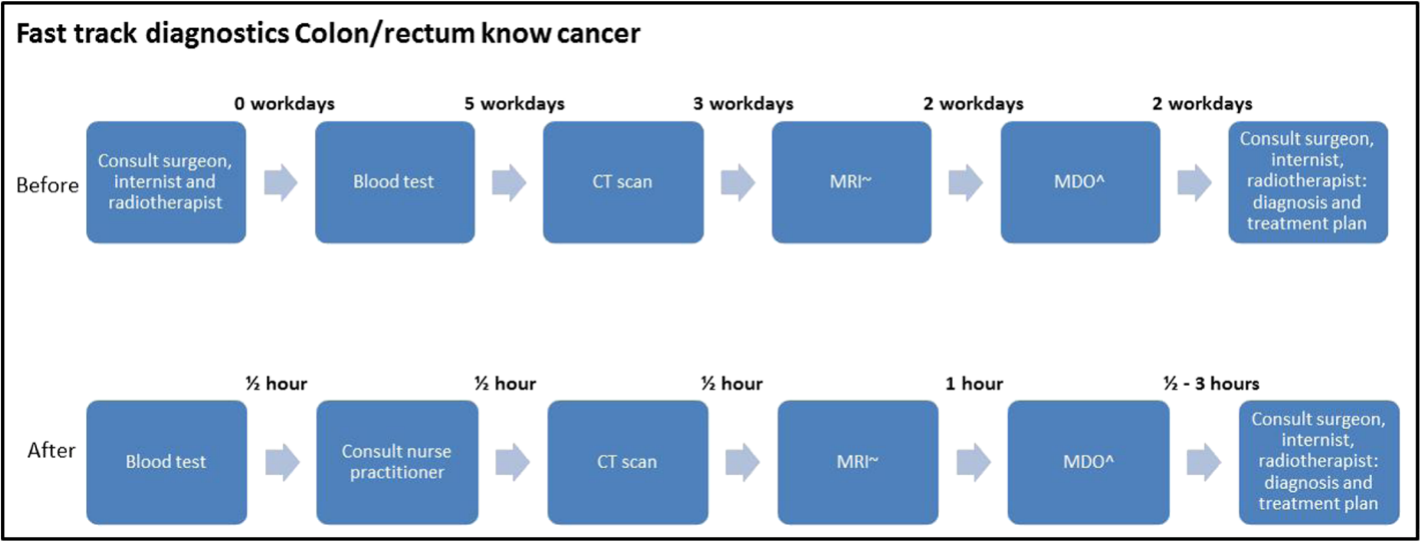
Mean throughput time in colon/rectum know cancer diagnostics before (measured total time, but guessed differentiated throughput time) and after fast track diagnostics implementation. ^MDO= multidisciplinary physicians meeting. ~MRI=magnetic resonance imaging

In Figs. 2 and 3 we provide two examples of process steps before and after the implementation with a time line. It proved thus possible to design 7 of 18 tumors as a ‘one stop shop’; a ‘shortest possible track’ for 4 cancer types related to examination under anesthesia (Head and Neck), a colonoscopy with preparation (colon/rectum cancer suspicion) or complex pathologic diagnostics (sarcoma and suspicious skin spot) and, based on patient reactions and related to MRI scheduling a 6 workday schedule for suspected prostate cancer. The latter was however a reduction with 8 workdays compared to the pre-redesign period. For 6 tumors we had to compromise to longer throughput times than necessary for medical reasons, for instance due to inability to change the moment of multidisciplinary team meeting for gynecological (max 7 workdays) and bladder tumors (6 workdays).

The implementation was planned in close contact with both the tumor boards and the diagnostic departments. A new pathology machine: all-in-one tissue processor, had to be acquired to enable same day pathology workup. With involvement of the radiology and nuclear medicine departments we succeeded in rearranging diagnostic PET, CT and MRI slots.

The start of fast track implementation for various cancer types was spread over a period of 5 months and required close monitoring of the project staff as a range of new procedures, process steps and communication routes were to be implemented at the same time. Solving practical issues, such as technical problems with diagnostic order-communication and coaching and supporting physicians and planners in the new process, proved a (time consuming) success factor.

Through involvement of clinical and board leadership, massive communication efforts through information sessions, intranet newsletters and internal media articles, the commitment of physicians to actually reschedule their weekly activities became rather positive.

In the post measurement after 3 and 6 months the mean access time was 8.2 (median 7) and 8.7 workdays (median 7) respectively and mean throughput time was 3.4 (median 1) and 3.3 workdays (median 1) respectively. The latter strongly influenced by the breast cancer numbers.

In Table 2 endometrium (*n* = 4), vagina (*n* = 1), vulva (n = 4), penis (*n* = 0) and testicle (*n* = 3) results were not presented due to small samples. However, also in these cancer types throughput time greatly decreased.

**Table 2:** Mean Throughput time (workdays) in post measurement

		After 3 months			After 3–6 months		
	Objective for 95%	Number of patients	Mean access time (workdays)	Mean throughput time (workdays)	Number of patients	Mean access time (workdays)	Mean throughput time (workdays)
Colon/Rectum cancer suspicion	3	7	8	3	7	6,7	3
Colon/Rectum known cancer	1	34	7,4	1	33	7,2	1
Cervix	7	7	6,5	4,3	4	10,8	4,5
Ovary	7	13	10,5	5	5	13	4,3
Breast	1	244	5,3	1*	247	5,1	1*
Head and Neck	11	77	7	7,6	56	7,7	9,5
Liver	1	19	7,6	1,2	29	7,8	1
Esophagus/stomach	1	11	7,7	1	30	7,3	1,3
Kidney	1	32	12,4	1	32	7,8	1
Bladder	6	42	10,6	6	49	9,2	5,3
Prostate	1 to 6^$^	132	12,7	4,5	135	16,2^	4
Sarcoma	7	45	8,7	11	40	8,8	11,6
Suspicious skin spot	6	16	11,3	6	12	7,8	6,5
Total		679	8,2	3,4	679	8,7	3,3

^$^Depending on if diagnosis with MRI is necessary

*Provisionally diagnosis, PA confirmation within 5 workdays

^^^Long access time due to patients who just had a biopsy elsewhere. For good imaging we need at least 6 weeks between biopsy and MRI

The actual use of slots was initially rather low compared to the prediction of the physicians, especially for CT and mostly in gynecological and esophagus/stomach tumors (Table 3). To prevent waste and negative impact on other patient groups, 48 h before the slot time, reserved capacity is made available for other groups. The radiology department reported that finally 90 to 100% of reserved capacity was thus used. After 6 months the match between available slots and actual use improved considerably for most fast track services. Matching slots and demand for gynecological and head and neck fast track services remained a challenge.

**Table 3:** Percentage of slot use of fast track reserved capacity after 6 months

	Slot use of fast track reserved capacity							
	After 3 months				After 6 months			
	MRI	CT	Echo	PET	MRI	CT	Echo	PET
Colon/Rectum known cancer^a^	54%	31%			54%	46%		
Cervix	16%	25%			12%	17%		
Endometrium								
Ovary								
Vagina								
Vulva								
Head and Neck	73%	46%	80%	67%	65%	32%	67%	47%
Liver				42%				57%
Esophagus/stomach^a^		25%	17%	33%		17%	33%	43%
Kidney^a^		23%	15%			31%	23%	
Bladder				67%				60%
Prostate	73%				89%			
Sarcoma	100%^b^	48%	29%		56% ^b^	75%	42%	

^a^Only one slot reserved, therefore less capacity is not possible

^b^Extra MRI capacity assigned in response to results after 3 months

Patient experiences were measured six months after implementation for a period of four weeks. During the measurement period, 107 patients received fast track diagnostics results. 97 questionnaires were sent and 63 were completed (response rate 65%). 10 patients were not able to receive emails. The overall score was 8.3 on a scale from 1 to 10.

A quarterly monitoring system was agreed upon to enable logistical adaptations on tactical planning level, such as re-dividing slots and enable management in acting on change requests. A staff that was involved in the project continued as a monitor/reporter and made suggestions, to adapt the systems tactical planning to recent developments and trends.

## Discussion

It proved possible to simultaneously design the process of 18 tumors as a fast track of which 7 as “one stop shop” (diagnosis completed in one visit). After 6 months of implementation mean access time was decreased from 10 to 15 to 8.7 workdays and mean throughput time was decreased from 6.0 to 3.3 workdays. In the second quarter after implementation, 27% of the eligible patients expressed the wish to receive fast track diagnostics.

In some tumor types, such as head and neck, speedy diagnostics and with that fast treatment start is likely to result in better treatment response. In other tumor types appointments about responsibilities in the diagnostics improved process control and the care quality.

The involvement of physicians in redesigning and reorganizing their work was important. The necessity for “change management” was addressed already in 1947 by Kurt Lewin [14], identifying relevant socio-dynamic forces and phases of introducing change. This approach was found applicable to change management projects in the hospital sector [15]. As especially personal adjustments in their weekly schedules and team conferences could occur, commitment of physicians and other stakeholders for change was considered essential. In order to build trust and unfreeze the views on the present situation, we let them actively participate in identifying problems and brainstorming on solutions within the group. This step was supported by communicating the results of the baseline measurement in staff meetings, rapid response to their needs and bottlenecks during implementation phase, supportive statements from senior clinical leadership and senior management and formal launch moments. External input from consultants was used to show relative value of existing practices.

Physicians became more open to think about the opportunities to create a better diagnostic process. We had to convince them that the status quo might not always be beneficial to their work by showing the value of alternative approaches such as central planning and the opportunity have a scan report in a few hours. To prevent blockage based on capacity worries, 4 h of additional MRI-capacity per week had to be decided upon as some senior physicians were convinced that some degree of redundancy would prove to be an essential success factor. This extra capacity was however not used in the reported period.

Some tumor groups tried to return to the old process using unforeseen implementation problems to argue that the new work process proved too difficult. Swift reacting to those ad hoc practical problems proved extremely useful.

Especially involvement of senior leadership in the decision process and a formal authorization procedure by team chair and pathway owner(s) is needed to inspire all stakeholders to “move”. A monitoring mechanism was introduced to control the changed processes and improve if necessary. During the follow up period, occasions to present the results were deliberately sought after to reinforce consistent implementation.

Research has shown that approaches such as operations research, lean management, six sigma and benchmarking, can help to improve patient logistics in healthcare [12, 13, 16, 17]. However for practicing clinicians, patient logistics appeared to be a rather new subject. Van Lent et al. 2012, showed that most Dutch hospitals used a combination of approaches and tools and top down steering was mostly absent; only about half of the hospitals reported goal accomplishment and no approach seemed to outperform the others [7]. In this project we noted that clinical staff is not so much interested in the type of operations management approach, but rather in the benefits that a project can generate for patients or staffs; it is thus not very usefull to accentuate the specific OM approach in communication efforts. Not just improving specific services, such as breast cancer, but a broad portfolio of services in a large scale project, requires operations knowledge to prevent suboptimization on organisational levels.

The belief that fast diagnoses relieve distress is the rationale supported by Leinster [18]. Contrary, Morse et al. [19], described women’s emotional responses when facing the possibility of breast cancer and conceptualize strategies for “getting through” the time between finding a breast lump, receiving news of an abnormal mammogram, and hearing biopsy results. They revealed ways that women cope an extremely distressing time in the diagnostic processes for breast cancer [19]. Enduring is a normal, natural, and even healthy response to a potential threat of an unavoidable loss that will continue until the person is able to cognitively accept the fact that they have (or may have) cancer. Further research should point out consequences of less waiting time and whether this is an important issue within fast track diagnostics, as we learned that a varying percentage per tumor type prefers not to enter the fast track procedure.

Another implication for further research is the impact of fast track diagnostics on health care costs. Although improving patient logistics is likely to reduce health care costs and lead to better use of infrastructure, this was not the objective of the project and no budgetary consequences were involved. The absence of a financial target may have assured physicians of the aligned, patient centered motives of senior management.

### Research limitations

Using a pre- post measurement design is a limitation of this study; however using a controlled design to improve the level of evidence in organizational improvement is very difficult to achieve [20].

It was a problem to trace the exact date of the diagnosis conversation in our patient files. This consultation is planned before the actual hospital visit and not always rescheduled in an identifiable way when complementary tests are necessary. In pre measurement it was not specifically registered, we therefore made assumptions to calculate the throughput time. Similar, the exact moment of referral was not registered in pre measurement, we therefore made assumptions to calculate the access time.

This study reports about implementation in a cancer center, with a lot of tertiary referrals. For generalization it is important to note that sufficient numbers per suspected tumor are needed to enable large scale redesign. Generalization possibilities will further depend on the degree of multidisciplinary cooperation and local financing conditions. Comparing larger series of implementations would shed light on their relative importance.

## Conclusions

It proved possible to redesign and implement fast track diagnostics of 18 cancer types within one year. Throughput time and access time were considerably shortened after implementation. The involvement of physicians in redesigning and reorganizing their work was a crucial success factor.

## Abbreviations

CT: Computed Tomography
MRI: Magnetic Resonance Imaging
NKI: Netherlands Cancer Institute
PET: Positron Emission Tomography
